# MRI appearance of ovarian serous borderline tumors of the micropapillary type compared to that of typical ovarian serous borderline tumors: radiologic-pathologic correlation

**DOI:** 10.1186/s13048-018-0379-y

**Published:** 2018-01-10

**Authors:** Go Nakai, Takashi Yamada, Kazuhiro Yamamoto, Yoshinobu Hirose, Masahide Ohmichi, Yoshifumi Narumi

**Affiliations:** 1 Department of Radiology, Osaka Medical College, 2-7 Daigaku-machi, Osaka, Takatsuki 569-8686 Japan; 2 Department of Pathology, Osaka Medical College, 2-7 Daigaku-machi, Osaka, Takatsuki 569-8686 Japan; 3 Department of Obstetrics and Gynecology, Osaka Medical College, 2-7 Daigaku-machi, Osaka, Takatsuki 569-8686 Japan

**Keywords:** Serous borderline tumor, Micropapillary, MRI, Imaging

## Abstract

**Background:**

Serous borderline tumor (SBT) of the micropapillary type (SBT-MP) became one of the major pathological SBT diagnoses in addition to typical SBT, and was also defined as “non-invasive” low-gradeserous carcinoma according to the World Health Organization (WHO) classification in 2014. In this study, we investigated the MRI appearance of SBT-MP compared to that of typical SBT in order to identify specific imaging features of SBT-MP that correspond to pathological findings.

**Methods:**

MR images of 6 histologically proven ovarian SBT-MP in four patients and 14 typical SBT in ten patients were reviewed retrospectively. Images were evaluated for laterality, size and morphology of the lesion and the solid component (SC) and signal intensity (SI) of the SC. MRI findings were correlated with pathological findings.

**Results:**

The patients with SBT-MP (mean 26.3 years) were younger than those with typical SBT (mean 44.5 years). Postoperative staging in patients with SBT-MP was II in two and III in two cases, while staging for typical SBT was I in seven, II in one and III in two cases. The morphologic patterns of SBT-MP were a unilateral cystic mass with intracystic mural nodules (CwMN) (*n* = 2), bilateral solid papillary masses (SM), and bilateral SM with CwMN. The pattern of typical SBT was CwMN (*n* = 13) in all but one lesion (SM with CwMN). All SCs showed inhomogeneous slight hyperintensity on T2 weighted images (WI) and high SI on diffusion-WI (DWI) except for in one typical SBT. Although diffuse proliferation of the tumor cells in micropapillary projections with little stroma seemed to correspond to inhomogeneous slightly hyperintense foci in SC on T2WI and high SI on DWI, similar MR findings were observed in typical SBT in all lesions on T2WI and 11 of 12 lesions on DWI. In typical SBT, inhomogeneous slightly hyperintense foci in SC on T2WI and high SI on DWI corresponded to highly cellular foci with densely branched papillae.

**Conclusion:**

Pathological findings and clinical behavior of SBT-MP differed from those of typical SBT, but morphology and SI of SC on MRI were similar, with papillary projections demonstrating inhomogeneous slight hyperintensity on T2WI and high SI on DWI.

## Background

Ovarian serous adenocarcinoma, which was defined as a malignant serous tumor according to the previous World Health Organization (WHO) classification in 2003 [1], was divided into two types, low-grade serous carcinoma (LGSC) and high-grade serous carcinoma, by the revised WHO classification in 2014 [2]. Serous borderline tumor (SBT) of the micropapillary type (SBT-MP) became one of the major pathological SBT diagnoses in addition to typical SBT, and was also defined as “non-invasive” LGSC. Several imaging reports regarding SBT have been published to date [3–5]. However, those cases might not be strictly divided into typical SBT and micropapillary type SBT because the previous WHO classification classified SBT into papillary cystic tumors, surface papillary tumors and adenofibromas, and cystadenofibromas primarily on the basis of macroscopic tumor cell proliferation as the major pathological diagnoses and did not include the micropapillary type until 2014.

Although SBT-MP account for only a small proportion of all SBT (10–15%), they exhibit more aggressive clinical behavior including invasive peritoneal implants and increased incidence of recurrence, which gives them a worse prognosis than that of typical SBT [6, 7]. Hence, it is important to distinguish between typical SBT and SBT-MP because aggressive surgical staging and debulking surgery may be recommended for SBT-MP.

MRI features specific to SBT-MP have never been investigated in detail. In this study, we investigated the MRI appearance of SBT-MP compared to that of typical SBT in order to identify specific imaging features of SBT-MP that correspond to pathological findings.

## Methods

Study subjects were selected from the four patients with SBT-MP and 20 patients with typical SBT who underwent surgery at our hospital between June 2009 and December 2016. Ten patients with typical SBT were excluded due to lack of availability of preoperative MR images (*n* = 4), collision tumor of the ovary associated with teratoma (*n* = 1), adnexal torsion (*n* = 1), rupture (*n* = 1) and recurrence (*n* = 3). Consequently, four patients with SBT-MP and ten patients with typical SBT were ultimately included in the study. Informed consent for clinical use of clinical data was obtained from each patient. The requirement for institutional review board approval was waived. Age and surgical staging were determined from medical records. MRI was performed with a 3.0-T superconducting magnet (Signa HDxt 3.0 T; GE Healthcare, Milwaukee WI, USA or 3 T MAGNETOM Skyra; Siemens Healthcare, Erlagen, Germany) for six patients (case 3 with SBT-MP and cases 3, 4, 5, 8 and 9 with typical SBT) and with a 1.5-T superconducting magnet (Signa HDxt; GE Healthcare, Milwaukee, WI or Achieva, Philips Healthcare, Best, Netherlands or MAGNETOM Symphony; Siemens Healthcare, Erlagen, Germany) using phased array coil for the others.

T2 weighted images (WI) were acquired in axial and sagittal planes. The scanning parameters for T2WI at 1.5 T [at 3.0 T] were as follows: TR (repetition time) range/TE (echo time) range, 3494–5117/90–108 [3750–6315/52–103]; slice thickness, 5–7 mm [5–6 mm]; interslice gap, 1–2 mm [1–2 mm]; matrix, 192×256–276×400 mm [224 × 320–410 × 512]; FOV, 25–36 cm [26–36 cm]. Axial T1WI were acquired with a spin-echo [enhanced T1 high-resolution isotropic volume examination, eTHRIVE] TR range/TE range of 115–800/4.2–13 [3.85/1.92], a 5–7 mm [5 mm] slice thickness/1 mm [0 mm] interslice gap, and a 256×192–250×384 mm ] [224–244×304–320 matrix. For all patients except one (case 2 with typical SBT), diffusion-weighted (DW) images were acquired in the axial plane using the single-shot echo-planar technique. The scanning parameters at b values of 0 and 800 or 1000 [1000] s/mm^2^ were as follows: 2935–7500/65–70 [4700–6777/64–72]; slice thickness, 5–6 mm [5–6 mm]; interslice gap, 0–1 mm [0–1.2 mm]; matrix,144×112–128×192 mm [112×84–192×128]; FOV, 36–40 [36]cm; NEX, 2–12 [1–5]. Diffusion-encoding gradients were applied along the three orthogonal directions of motion-probing gradients. For ten patients (cases 1 and 4 with SBT-MP and all cases with typical SBT with the exception of cases 6 and 9), gadoxetic acid-enhanced images were obtained by fat-suppressed T1WI using the spectral attenuated inversion recovery (SPAIR) technique (Achieva, Philips Healthcare: 575–641/13; matrix, 288 × 201 mm; 5-mm section thickness and 25-cm field) or T1 weighted 3D sequence (liver acquisition with volume acceleration [LAVA] using the spectral presaturation with inversion recovery [SPIR] technique) (GE Healthcare: 3.37–3.65/1.74–2.07; flip angle, 12°; matrix, 256–320 × 192–224 mm; 5–6 mm section thickness and a 26–30 cm field). A 0.01 mmol/kg body weight dose of contrast agent was administered intravenously followed by a 20-ml saline flush.

MR images were analyzed by G.N., who has 12 years of experience in gynecological imaging. The MRI features of the tumors were assessed with respect to six categories: (1) laterality, (2) size and morphology, (3) septation of cystic components (CC), (4) size of the largest solid component (SC), (5) signal intensity (SI) of the largest SC and (6) the enhancement pattern.

Size and morphology was classified as (i) cystic with intracystic mural nodules (CwMN), (ii) solid, or (iii) mixed CwMN-solid. A nodule was defined as a solid component with a maximum diameter less than 3 cm. SI of the SC on T2WI was classified as “hyperintense” if the SI was equal to that of water, “hypointense” if it was equal to that of muscle, and “slightly hyperintense” if it was between hyperintense and hypointense. SI of the CC on T1WI was classified as “hypointense” if it was equal to that of water, “hyperintense” if it was equal to that of fat, and “slightly hyperintense” if it was between hyperintense and hypointense. On DWI, the SI of the SC was classified as “hyperintense” if the SI was equal to that of uterine endometrium, “hypointense” if it was equal to that of muscle, and “slightly hyperintense” if it was between hyperintense and hypointense.

Contrast enhancement was considered “intense” when the SI of the lesion was equal to that of uterine myometrium.

The amount of ascites was classified as small when it was limited to the pouch of Douglas, moderate when it was observed above the level of the uterine fundus, and large when abdominal distention due to ascites was observed. Peritoneal implantation was considered to have occurred when any nodule was observed in the intraperitoneal space. The MRI findings were correlated with pathological findings by a gynecological pathologist (T.Y.) with 30 years of experience.

Statistical analyses were performed using the Wilcoxon rank sum test to confirm the difference in the maximum diameter of the tumor and the largest solid component between SBT-MP and typical SBT. The difference was regarded as significant when *p*-value was less than or equal to 0.05.

## Results

Age, laterality, size and morphology of lesions are summarized in Table 1. The age ranges for SBT-MP and typical SBT were 25–27 years (mean, 26.3 years) and 25–71 years (mean, 42.3 years), respectively. Two of four patients with SBT-MP and four of ten patients with typical SBT had bilateral ovarian involvement. Fertility-sparing surgery was performed for two patients with SBT-MP and five patients with typical SBT, and standard ovarian borderline tumor surgery including total hysterectomy, bilateral salpingo-oophorectomy, and omentectomy was performed for the others. The stage of SBT-MP was IIa in two patients, IIIa in one patient, and IIIb in one patient, while that of typical SBT was Ia in five patients, Ib in one patient, Ic in one patient, IIc in one patient and IIIa in two patients. All six SBT-MP included mural nodules or solid components like typical SBT. The maximum diameter of SBT-MP ranged from 43 to 76 mm (mean, 62 mm), while that of typical SBT ranged from 22 to 174 mm (mean, 72.6 mm) (*P* = 0.96). The mean maximum diameter of the largest mural nodule or solid component of SBT-MP was 37.2 mm (range 4–59 mm), while that of typical SBT was 23.6 mm (range 7–59 mm) (*P* = 0.18).

**Table 1:** Age, laterality, size and morphology of lesions in patients with ovarian SBT-MP and typical SBT

	SBT-MP (*n* = 4)	Typical SBT (*n* = 10)	*P* value*
The mean age (range)	26.3 (25–27)	42.3 (25–71)	
Laterality			
Unilateral	2	6	
Bilateral	2	4	
Shape of the mass			
Solid	2	0	
CwMN	2	13	
Mixed CwMN-Solid	2	1	
Septation of cystic mass	4/4	8/14	
MD of the tumor (range) (mm)	62 (43–76)	72.6 (22–174)	0.96
MD of the largest SC (range) (mm)	37.2 (4–59)	23.6 (7–59)	0.18

*CwMN* cystic mass with intracystic mural nodules, *MD* maximum diameter

*Results from Wilcoxon rank sum test

**Fig. 1:**
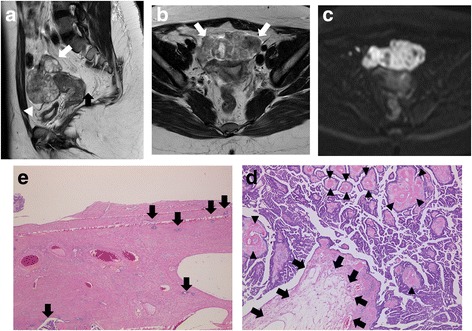
Case 3 with SBT-MP. 25-year-old woman with bilateral ovarian SBT-MP. Sagittal T2WI (**a**) shows a solid mass (arrowhead) with inhomogeneous slight hyperintensity on the surface of a normal left ovarian structure (white arrow). The contralateral mass showed similar findings of normal ovarian structure within the mass (not shown). A small amount of ascites is present (black arrow). The internal branching architecture within both masses (arrows) is obscure on both sagittal and axial T2WI (**b**). Both ovarian masses are hyperintense on DWI (**c**). A photomicrograph (**d**) shows diffuse proliferation of the tumor cells in micropapillary projections with little stroma. Pinkish-stained hyaline degeneration (arrowheads) and randomly distributed edema (arrows) in the internal branching fibrous stalk are observed. Tiny cystic lesions that appeared deceptively normal on MRI were confirmed in both ovarian cortexes (**e**)

**Fig. 2:**
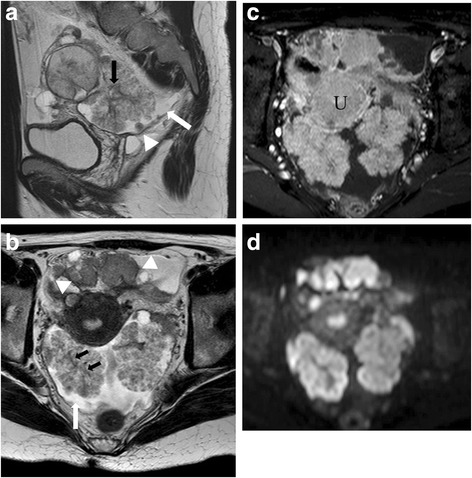
Case 4 with SBT-MP. 27-year-old woman with bilateral ovarian SBT-MP. Sagittal T2WI (**a**) shows left solid papillary masses on the surface of multicystic masses with intracystic mural nodules. On axial T2WI (**b**), bilateral solid papillary masses demonstrate inhomogeneous slight hyperintensity and its internal branching architecture is partially detected as an inhomogeneous low signal branch-like structure is detected on T2WI (black arrow, **a**, **b**). A moderate amount of ascites (white arrow, **a**, **b**) and peritoneal implants (arrowheads, **a**, **b**) are present. After administration of gadolinium DTPA, the solid masses and peritoneal implants were “intensely enhanced” with the SI of the lesion equal to that of uterine myometrium (U) (**c**). On DWI, both ovarian masses are hyperintense (**d**)

**Fig. 3:**
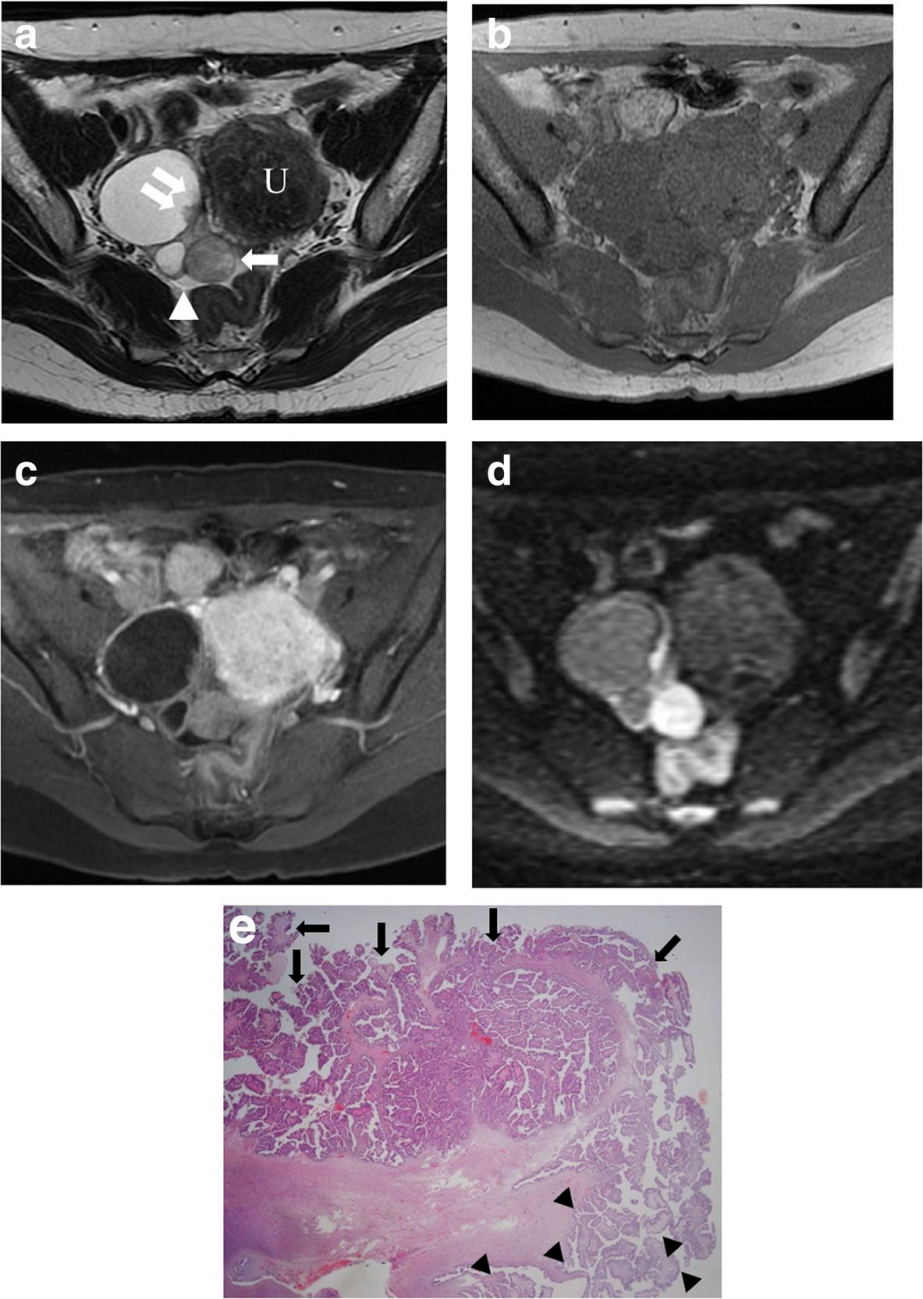
Case 1 with SBT-MP. 27-year-old woman with right ovarian SBT-MP. Axial T2WI (**a**) shows a multicystic mass on the right side of the uterus (U) with intracystic mural nodules showing inhomogeneous slight hyperintensity (arrows). Hypointense internal branching architecture is not detected. A small amount of ascites is noted (arrowhead). The mass shows homogeneous hypointensity on axial T1WI (**b**). The mural nodules are homogeneously enhanced after administration of gadolinium DTPA (**c**) and hyperintense on DWI (**d**). The corresponding histopathological section shows papillary architecture with a hierarchical internal branching fibrous stalk, with diffuse proliferation of tumor cells in elongated micropapillary projections with little or no stromal support peripherally (arrows). Whereas the co-existing typical SBT has a peripheral edematous stalk with superficial epithelial cells, a loose structure of papillary projections is noted in the same papillary projection (arrowheads) (**e**)

**Fig. 4:**
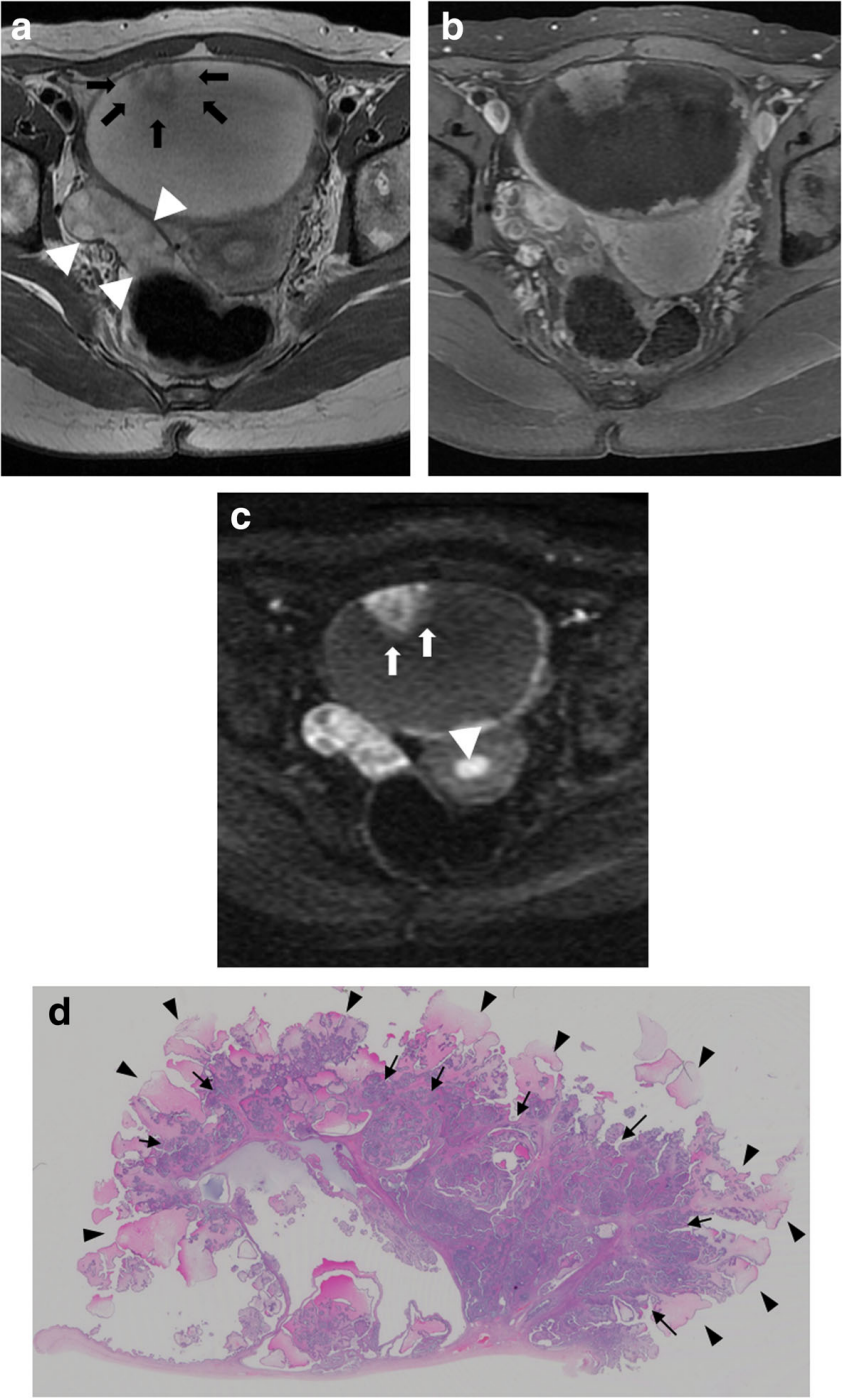
Case 3 with typical SBT. 25-year-old woman with left ovarian typical SBT. Axial T2WI (**a**) shows a left ovarian cystic mass with intracystic mural nodules. The largest papillary nodule (arrows) demonstrates inhomogeneous slight hyperintensity in the internal portion and hyperintensity equal to SI of cystic content in the external portion. The normal right ovary is present (arrowheads). After administration of gadolinium DTPA, the mural nodules are enhanced and the conspicuity is increased (**b**). On DWI, the internal portion of the largest papillary nodule shows hyperintensity equal to the SI of uterine endometrium (arrowhead) but the external portion (arrows) shows less signal intensity. **c** A photomicrograph of the area corresponding to the largest papillary nodule (**d**) shows that foci with densely branched papillae with little space among papillae are frequently found in the internal portion (arrows), corresponding to inhomogeneous slight hyperintensity on T2WI. In contrast, a loose papillary architecture of peripheral vegetation with edema (arrowheads) tends to be observed in the external portion, corresponding to hyperintensity on T2WI

**Fig. 5:**
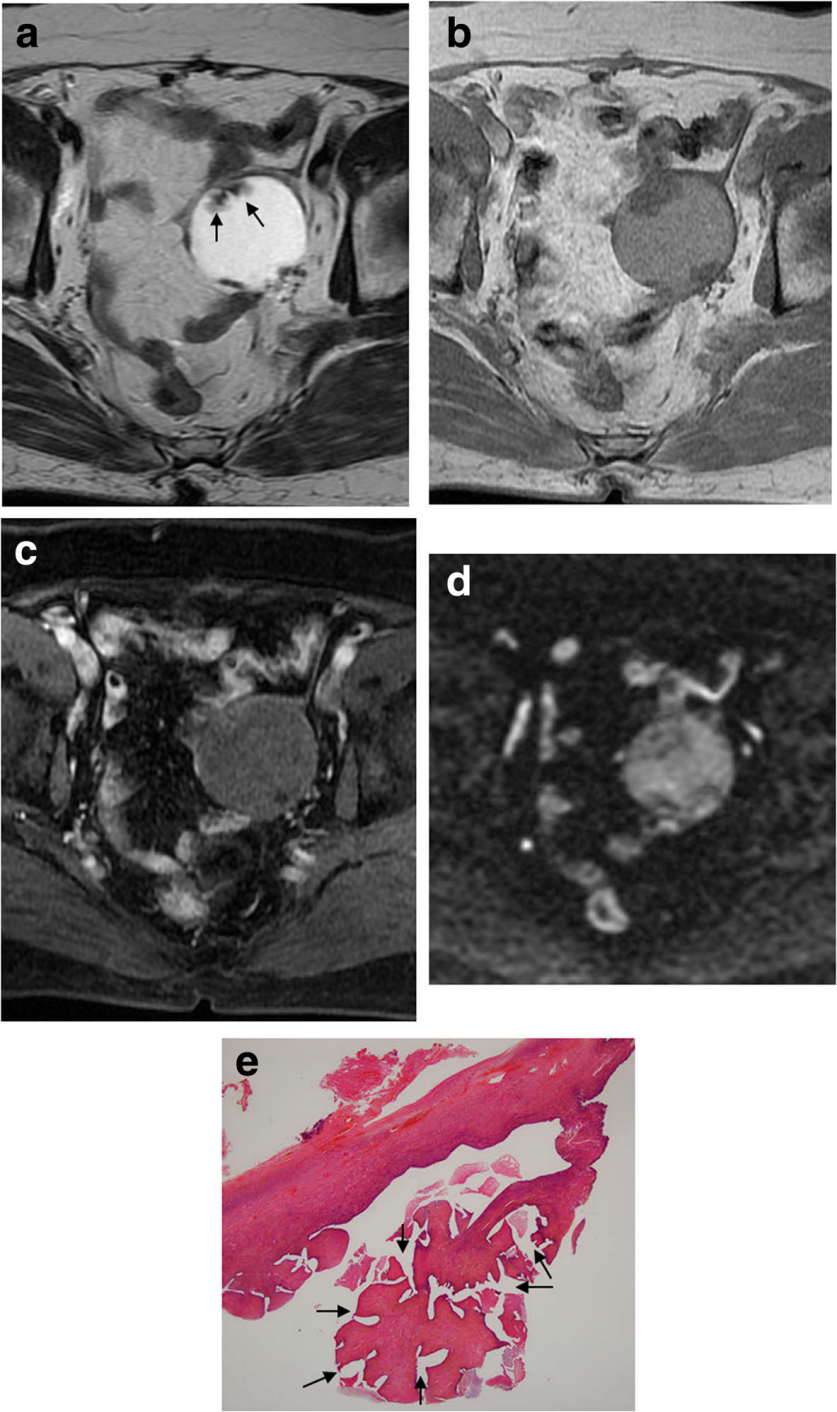
Case 7 with typical SBT. 65-year-old woman with left ovarian typical SBT. Axial T2WI (**a**) shows a left ovarian cystic mass with intracystic mural nodules. The largest papillary nodule (arrows) demonstrates inhomogeneous slight hyperintensity with internal hypointense foci. On T1WI (**b**), the SI of the cystic content shows slight hyperintensity. After administration of gadolinium DTPA (**c**), the mural nodules are enhanced weakly. On DWI (**d**), the largest mural nodule shows hypointensity equal to the SI of muscle. A photomicrograph of the area corresponding to the largest papillary nodule (**e**) shows a bluntly branched fibrous stalk without edema and hyaline degeneration. More spaces between papillae are found due to the lack of densely branched papillae peripherally (arrows), leading to hypointensity on DWI

The MRI appearance of SBT-MP and typical SBT is summarized in Table 2. The morphologic pattern was bilateral tumors with a solid component on the surface of the normal ovarian structure (Fig. 1) in one patient with SBT-MP, bilateral tumors with solid components on the surface of a multicystic mass with intracystic mural nodules (Fig. 2) in another patient with SBT-MP, and a unilateral tumor with those features in one patient with typical SBT. The other lesions showed a multicystic mass with intracystic mural nodules (Fig. 3). Two of four CC of SBT-MP and four of 14 CC of typical SBT were slightly hyperintense on T1WI and hyperintense on T2WI, and the others were hypointense on T1WI and hyperintense on T2WI. The quality of CC in four of six cystic lesions which showed slight hyperintensity or hyperintensity on T1WI was mentioned in clinical records. Serous fluid was confirmed in two lesions and whitish mucinous fluid in the others. All mural nodules had a nodular shape and all SC had a papillary shape. All mural nodules and SC of SBT-MP showed inhomogeneous slight hyperintensity on T2WI and hyperintensity on DWI. Although SC of typical SBT also showed inhomogeneous slight hyperintensity on T2WI and hyperintensity on DWI, five lesions included hyperintense foci in their SC on T2WI (cases 2, 3, 4 and 10) (Fig. 4) and one lesion (case 7) showed hypointensity on DWI (Fig. 5). After administration of Gd-DTPA (gadolinium-diethylenetriamine pentaacetic acid), mural nodules and SC of the bilateral tumors in one patient with SBT-MP and (case 4; Fig. 2) and mural nodules of the unilateral mass in one patient with typical SBT were intensely enhanced. Mural nodules and SC of the other lesions were enhanced. A small amount of ascites was observed in three patients with SBT-MP and seven patients with typical SBT, a moderate amount in one with typical SBT, and a large amount in one with SBT-MP. Peritoneal dissemination was obvious in one patient with SBT-MP (case 4; Fig. 2) and two patients with typical SBT (cases 2 and 4) but not detected in the others.

**Table 2:** MRI appearance of SBT-MP and typical SBT

Case	Age	Affected side	Shape of the mass	The SI of CC (T1WI/T2WI)	The SI of MNs or SCs on T2WI	Homogeneity in SI of MNs or SCs on T2WI	The SI of the largest MN or SC on DWI	Contrast enhancement of MNs or SCs	Peritoneal implant	Ascites	Stage
SBT-MP											
1	27	right	CwMN	hypo/hyper	slightly hyper	inhomogeneous	hyper	enhanced	–	small	IIa
2	26	left	CwMN	hypo/hyper	hypo-slightly hyper	inhomogeneous	hyper	NA	–	small	IIa
3	25	right	Solid	NA	slightly hyper	inhomogeneous	hyper	NA	–	small	IIIa
		left	Solid	NA	slightly hyper	inhomogeneous	hyper	NA			
4	27	right	Mixed CwMN-solid	slightly hyper/hyper	slightly hyper	inhomogeneous	hyper	intensely enhanced	+	large	IIIc
		left	Mixed CwMN-solid	slightly hyper/hyper	slightly hyper	inhomogeneous	hyper	intensely enhanced			
Typical SBT											
1	54	right	CwMN	hypo/hyper	slightly hyper	homogeneous	hyper	enhanced	–	small	Ia
2	32	right	Mixed CwMN-solid	slightly hyper/hyper	slightly hyper-hyper	inhomogeneous	NA	enhanced	+	moderate	IIc
		left	CwMN	slightly hyper/hyper	slightly hyper	inhomogeneous	NA	enhanced			
3	25	left	CwMN	hypo/hyper	slightly hyper-hyper	inhomogeneous	hyper	enhanced	–	small	Ia
4	43	right	CwMN	slightly hyper/hyper	slightly hyper-hyper	inhomogeneous	hyper	enhanced	+	small	IIIa
		left	CwMN	hypo/hyper	slightly hyper-hyper	inhomogeneous	hyper	enhanced			
5	33	right	CwMN	hypo/hyper	slightly hyper	homogeneous	hyper	enhanced	–	small	Ic
6	53	right	CwMN	hypo/hyper	slightly hyper	homogeneous	hyper	NA	–	none	Ia
7	65	left	CwMN	slightly hyper/hyper	hypo- slightly hyper	inhomogeneous	hypo	weakly enhanced	–	none	Ia
8	34	right	CwMN	hypo/hyper	slightly hyper	inhomogeneous	hyper	intensely enhanced	–	small	Ia
9	25	right	CwMN	hypo/hyper	slightly hyper	inhomogeneous	hyper	NA	–	small	Ib
		left	CwMN	hypo/hyper	slightly hyper	inhomogeneous	slightly hyper-hyper	NA			
10	71	right	CwMN	hyper/hyper	slightly hyper-hyper	inhomogeneous	hyper	enhanced	–	small	IIIa
		left	CwMN	hypo/hyper	slightly hyper	inhomogeneous	hyper	enhanced			

*CC* cystic component, *NA* not assessed, *CwMN* cystic mass with intracystic mural nodules, *MD* maximum diameter, *SI* signal intensity, *MN* mural nodule, *SC* solid component, *hyper* hyperintensity, *hypo* hypointensity

Histopathological analysis of SBT-MP cases revealed that all mural nodules and solid components had a papillary architecture with a hierarchical internal branching fibrous stalk containing diffusely proliferating tumor cells in elongated micropapillary projections with little or no stromal support peripherally. Peripheral diffuse proliferation of the tumor cells in elongated micropapillary projections with little or no stromal support corresponded to areas of inhomogeneous slightly hyperintense foci on T2WI and hyperintense foci on DWI. In contrast, typical SBT lacked the peripheral diffuse proliferation of tumor cells in elongated micropapillary projections with little or no stromal support. Nevertheless, mural nodules and SC showing hyperintensity on DWI corresponded to highly cellular foci filled with densely branched papillae with little space among papillae. However, a scattered loose papillary architecture of peripheral vegetation with edema was observed in some cases, leading to hyperintense foci in solid components on T2WI and less signal intensity in those areas on DWI (case 3 with typical SBT; Fig. 4). Hyaline degeneration and edema within the proximal fibrous stalk were observed in both SBT-MP and typical SBT. These changes might make the stromal cellularity inhomogeneous and scanty, leading to the inhomogeneous hyperintensity on T2WI.

## Discussion

The mean age of patients with typical SBT is reported to be 42 years (range, 36.8 to 43 years), whereas that of patients with SBT-MP is 45 years, which is younger than patients with serous carcinoma. In our series the mean age was 26.3 years, which is much younger than patients with typical SBT or SBT-MP. Furthermore, the FIGO (International Federation of Gynecology and Obstetrics) stage of SBT-MP was stage II with noninvasive intrapelvic implants in two patients and stage III with noninvasive peritoneal implants in two patients. Considering that 65–80% of patients with typical SBT are in stage I, the stage of SBT-MP is more advanced than that of typical SBT, illustrating the aggressive feature. LGSC characterized by KRAS, BRAF, and ERBB2 gene mutations is thought to show stepwise progression and develop from serous cystadenomas and SBT [8–10]. In fact, histopathological analyses revealed transformation zones from typical SBT in several places in our series (Fig. 3e).

The current WHO classification defines SBT-MP as a non-invasive tumor with a nonhierarchical branching architecture featuring micropapillary and/or cribriform patterns composed of rounded cells with scant cytoplasm and moderate nuclear atypia [2]. Although the characteristic shape and SI of SBT-MP on MRI resembled typical SBT, the causes of the SI corresponded to different histopathological findings in this study. The inhomogeneous slight hyperintensity of papillary projections in patients with SBT-MP on T2WI corresponded to tumor cells proliferating densely as elongated thin micropapillae with little or no stromal support resulting in fewer spaces between papillary clusters. Although typical SBT lack elongated thin micropapillae with little or no stromal support, the foci with densely branched papillae with little space among papillae seemed to result in slight hyperintensity on T2WI and hyperintensity on DWI. However, a loose papillary architecture of peripheral vegetation with edema was observed in some mural nodules and solid components of typical SBT, leading to hyperintensity on T2WI and less signal intensity on DWI. Consequently, mural nodules and solid components of SBT-MP can show lower signal intensity with obscure internal branching architecture on T2WI and higher signal intensity on DWI than typical SBT. However, it is difficult to differentiate SBT-MP from typical SBT by referring to SI of mural nodules and SC on MRI. Furthermore, hyaline degeneration and edema within the fibrous stalk in both SBT-MP and typical SBT were randomly observed histopathologically, further contributing to the inhomogeneity in SI of mural nodules and SC on MRI. According to previous literature, the common MR finding of typical SBT is papillary projections that show high SI equal to water with a hypointense internal branching architecture on T2WI, intermediate SI on DWI, and enhancement after administration of Gd-DTPA [5, 11]. The hypointensity of the internal branching architecture on T2WI corresponds to the fibrous stalk histopathologically. In contrast, the high SI of papillary projections on T2WI and intermediate SI on DWI are attributed to the peripheral edematous stalk with superficial epithelial cells, the loose structure of papillary projections, and fluid permeating multiple spaces between papillary clusters [3–5, 11, 12]. In contrast, most papillary projections of typical SBT showed inhomogeneous slight hyperintensity on T2WI and hyperintensity on DWI in this study. The discrepancies in SI imply that SI of papillary projections may depend on the number of spaces between papillary clusters and the amount of loose papillary architecture of peripheral vegetation with edema (Figs. 4, 5). In fact, the spaces between papillary clusters of mural nodules and SC in typical SBT varied due to their shape, even within the same tumor. Furthermore, the internal fibrous stalk did not always show hypointensity on T2WI due to its hyaline degeneration and edema (Figs. 1, 2).

On fat-saturated gadolinium-enhanced T1WI, SBT-MP showed enhancement or intense enhancement with a slightly enhanced internal fibrous stalk. This enhancement pattern is similar to that of typical SBT, which indicates that although contrast-enhanced MRI may not be helpful in differentiating SBT-MP from SBT, it does increase the conspicuity of papillary projections, especially when they are small and endophytic (Fig. 4b) [13].

Basically, the SI of CC was equal to that of water, but two of four CC of SBT-MP and five of 14 CC of typical SBT showed slight hyperintensity or hyperintensity on T1WI. In previous literature, a yellow-green mucoid fluid in the CC corresponded to hyperintense signal on T1WI [5].

Bilateral tumors were observed in two of four patients with SBT-MP and four of ten patients with typical SBT. In case 2 with SBT-MP, the patient underwent left salpingo-oophorectomy and had a relapse at the contralateral ovary after 2 years and 7 months. The relapsed contralateral ovary included several tiny lesions in the cortex that were not detected preoperatively on MRI. In cases with bilateral ovarian involvement, it is not easy to distinguish whether one is primary or metastatic or both are primary, even by pathological analysis of resected specimens. However, many tiny cystic lesions that appeared deceptively normal on MRI were confirmed in both ovarian cortexes in case 3 with SBT-MP (Fig. 1). These findings suggest that SBT may be related to synchronous and metachronous disease. They also imply a causal connection that incorporation of fallopian tube epithelium into ovarian cortical inclusion cysts during ovulation may also be the cause of low-grade serous neoplasms as well as high grade serous carcinoma. Therefore, radiologists should carefully evaluate both ovaries when one ovary seems to be SBT on MRI.

This study has some limitations. Due to its retrospective nature and the small number of SBT-MP cases (*n* = 4), further investigation using MRI on more patients with SBT-MP is warranted.

## Conclusions

Pathological findings and clinical behavior of SBT-MP differed from those of typical SBT, but their morphology and SI of SC on MRI were similar, with papillary projections demonstrating inhomogeneous slight hyperintensity on T2WI and high SI on DWI.

## Abbreviations

CA: carbohydrate antigen
CC: cystic component
CEA: carcinoembryonic antigen
CwMN: cystic mass with intracystic mural nodules
DWI: diffusion-weighted images
eTHRIVE: enhanced T1 high-resolution isotropic volume examination
FIGO: International Federation of Gynecology and Obstetrics
Gd-DTPA: gadolinium-diethylenetriamine pentaacetic acid
LAVA: liver acquisition with volume acceleration
LGSC: low-grade serous carcinoma
MRI: magnetic resonance imaging
SBT: serous borderline tumor
SBT-MP: serous borderline tumor of the micropapillary type
SC: solid component
SI: signal intensity
SM: solid papillary masses
SPAIR: spectral attenuated inversion recovery
SPIR: spectral presaturation with inversion recovery
T: tesla
TE: echo time
TR: repetition time
WHO: World Health Organization
WI: weighted images
